# Evolutionary impact of copy number variation rates

**DOI:** 10.1186/s13104-017-2741-3

**Published:** 2017-08-10

**Authors:** Guillermo Rodrigo

**Affiliations:** Institute for Integrative Systems Biology, CSIC-UV, 46980 Paterna, Spain

**Keywords:** Birth–death process, Gene duplication, Genome size, Neutral evolution, Population genetics

## Abstract

**Objective:**

Copy number variation is now recognized as one of the major sources of genetic variation among individuals in natural populations of any species. However, the relevance of these unexpected observations goes beyond diagnosing high diversity.

**Results:**

Here, it is argued that the molecular rates of copy number variation, mainly the deletion rate upon variation, determine the evolutionary road of the genome regarding size. Genetic drift will govern this process only if the effective population size is lower than the inverse of the deletion rate. Otherwise, natural selection will do.

## Introduction

The advent of genomic systems biology is leading, in very recent years, to the discovery of widespread genetic features, previously unrecognized in complex organisms like humans; they can then have strong implications in biomedicine. One of these fascinating features is copy number variation [1], which is already considered one of the major sources of genetic variation. Thereby, in natural populations, some individuals have significant portions of the genome repeated, even entire genes, something until now believed to occur at a large scale only in microbes [2]. Recently, genomic studies with populations of different model organisms are serving to estimate copy number variation rates [1, 3], revealing great differences among them. However, the generation of genetic diversity, here genome rearrangements, cannot be fully understood without accounting for an evolutionary perspective [4]. In this regard, what is the impact of these rates?

In this short piece, it is argued that these rates greatly determine the way by which the genome can increase its size, i.e., the evolutionary force that controls this process. Indeed, the acquisition of genetic redundancy is believed to be the major mechanism to increase genome size, and then genome complexity [5, 6]. For that, duplications have to be fixed in the population, a process that mainly occurs, according to the classical theory, by random genetic drift under effectively neutral selective conditions thanks to a reduction in effective population size [6]. However, the balance between duplication and deletion, at a given locus, regulates the power of drift in fixation, as it is illustrated here with a simple quantitative analysis.

## Main text

### Theory

The complex process of copy number variation in a single organism, which involves widely different mechanisms [7], can be simplified to a birth–death process. This allows creating a toy model from which to make predictions (see “Limitations”). If *μ* denotes the duplication rate of a locus and *λ* the deletion rate upon duplication, the frequency of the genotype with two copies (*x*) in a population of size *N* is governed by the following stochastic differential equation

1 $$\frac{dx}{dt} = \mu - (\mu + \lambda )x + \sqrt {\frac{{x\left( {1 - x} \right)}}{N}} z\left( t \right) ,$$

where *t* is measured in generations, and *z*(*t*) is a stochastic process with mean zero and correlation delta, having assumed a Wright-Fisher reproduction model and strictly neutral selective conditions [8]. This means that the stationary solution will be *x* = *μ*/(*μ* + *λ*), and that the eventual fixation of genetic variants will only occur transiently. This is an important consideration, implying that duplicates will be preserved for long time if they quickly accumulate, upon fixation, beneficial [9] or complementary, degenerative mutations [10], escaping from the birth–death process.

The system has two different time scales, one given by 1/(*μ* + *λ*), associated with copy number variation, the other by *N*, associated with genetic drift. Certainly, if (*μ* + *λ*)*N* ≪ 1, the system can be assumed dominated by genetic drift at short times. Accordingly, the typical fluctuation amplitude in frequency (Δ*x*, around the stationary solution) can follow the Einstein’s theory of Brownian particles [11]. A fixation time of *t* ≈ 6*N* can be derived if we integrate over [0, 1] the variance of the stochastic process, *x*(1 − *x*)/*N*, to have constant diffusion, in good tune with the Kimura’s calculation [8]. But, in general, we have

2 $$\Delta x = \frac{1}{{\sqrt {12(\mu + \lambda )N} }} .$$

Fixation will occur in displacements that reach *x* = 1 [i.e., Δ*x* = *λ*/(*μ* + *λ*)], which yields the condition of *λN* < 1/12, when *λ* ≫ *μ* (typically in nature) [1]. Fluctuations can even be three times the typical value, although they will occur sporadically. This yields the soft condition of *λN* < 1 to have chances for fixation. By contrast, if (*μ* + *λ*)*N* ≫ 1, the system is mostly dominated by the balance between duplication and deletion. Therefore, Δ*x* ≪ 1, which entails that duplications cannot be fixed.

### Remark

The deletion rate that was considered here is the rate at which a repeated portion of the genome is deleted. Certainly, duplication imposes a genetic instability that is generally resolved by deletion [12]; sometimes by other means, like relocation [13]. Experimentally, such a deletion rate needs to be estimated from populations with individuals carrying duplications. The deletion rate of significant, but unique fragments is expected to be only a lower bound. Despite, this has already been proposed as a determinant of genome size [14].

### Application

**Fig. 1:**
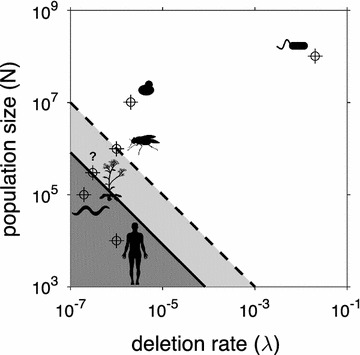
Phase space between effective population size (*N*) and deletion rate upon duplication (*λ*) showing the region where genome size can increase by genetic drift (*shaded region*). The *solid line* corresponds to the limit *λN* = 1/12 (strict), whilst the *dashed line* to *λN* = 1 (soft). *λ* ≫ *μ* is assumed. Six different organisms are contextualized (*S. enterica*, *S. cerevisiae*, *D. melanogaster*, *C. elegans*, *A. thaliana*, and *H. sapiens*). All *λ* values correspond to empirical estimations, except the value for the plant, which is a prediction

This simple theory can be applied to analyze the fixation ability in different organisms (Fig. 1). For *Salmonella enterica*, *μ* ≈ 10^−4^/locus/gen. and *λ* ≈ 2·10^−2^/locus/gen. [12], with *N* ≈ 10^8^. In this case, (*μ* + *λ*)*N* ≈ *λN* ≈ 2·10^6^ ≫ 1, which entails that this bacterium cannot acquire genetic redundancy, at least by drift. Similar is the case for the lower eukaryote *Saccharomyces cerevisiae*, where *μ* ≈ 3·10^−6^/locus/gen. and *λ* ≈ 2·10^−6^/locus/gen. [3], with *N* ≈ 10^7^, give (*μ* + *λ*)*N* ≈ 50 ≫ 1. However, the scenario is different in higher eukaryotes. For *Drosophila melanogaster*, *μ* ≈ 2·10^−7^/locus/gen. and *λ* ≈ 10^−6^/locus/gen. [15], with *N* ≈ 10^6^. This results in (*μ* + *λ*)*N* ≈ *λN* ≈ 1, the soft limit, suggesting that transient fixation of duplications could occur. Better is the case for *Caenorhabditis elegans*, as *μ* ≈ 10^−7^/locus/gen. and *λ* ≈ 2·10^−7^/locus/gen. [16], with *N* ≈ 10^5^, lead to (*μ* + *λ*)*N* ≈ 0.03 ≪ 1. For *Homo sapiens*, *μ* + *λ* ≈ 10^−6^/locus/gen. [1], with *N* ≈ 10^4^, ensures many momentary fixations by drift, as (*μ* + *λ*)*N* ≈ 0.01 ≪ 1.

But the rates (*μ* and *λ*) confidently estimated until now (in mutation accumulation experiments) are really scarce, only available for some model organisms [17]. In addition, the deletion rates might be underestimated (see “Remark”). For *Arabidopsis thaliana*, e.g., only bioinformatic estimates have been produced, although these give values that differ from experimental estimates in several orders of magnitude. Based on the values of *D. melanogaster* and *C. elegans*, one can predict *λ* ≈ 10^−7^–10^−6^/locus/gen. for *A. thaliana*, resulting in *λN* ≈ 0.03–0.3 < 1, as *N* ≈ 3·10^5^. Higher eukaryotes have indeed more chances to transiently fix duplications by drift due to a reduced effective population size [6].

### Conclusion

Definitely, *λN* < 1 has to be satisfied in order to reach transient fixation of duplications by genetic drift. Otherwise, the population remains stably polymorphic regarding copy number. If this were the case, positive selective conditions should be invoked to explain an increase in genome size. After all, the precise characterization at the molecular level of the genome rearrangement rates, especially the deletion rate upon duplication, will shed much light to recognize how fortuitous was the path to reach the life that today we see on the Earth [18].

## Limitations

The following limitations associated with the mathematical model were identified:

- Simplification to a birth–death process, while genome rearrangements may be more complex processes (e.g., gene relocation in the chromosome to stabilize a duplicate).
- No consideration of high-order variations, such as gene triplications or quadruplications, while these are found in nature. No consideration of individuals with zero copies, assuming they are deleterious and then quickly diluted.
- Population size assumed constant, while this may vary with time due to multiple environmental factors.
